# Supplementary material for: SsCak1 Regulates Growth and Pathogenicity in Sclerotinia sclerotiorum
Source: Int J Mol Sci. 2023 Aug 9;24(16):12610. doi: 10.3390/ijms241612610 (PMC10454577; doi:10.3390/ijms241612610)
Supplement: Supplementary file 1 [file ijms-24-12610-s001.zip › ijms-2540454-SI.pdf]

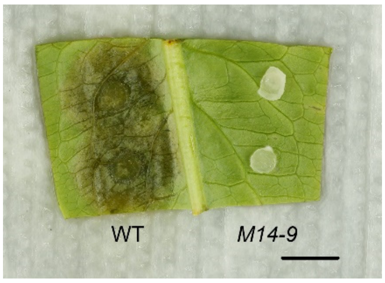

Figure S1: Identification of a pathogenicity-deficient mutant M14-9 by screening on lettuce leaves.

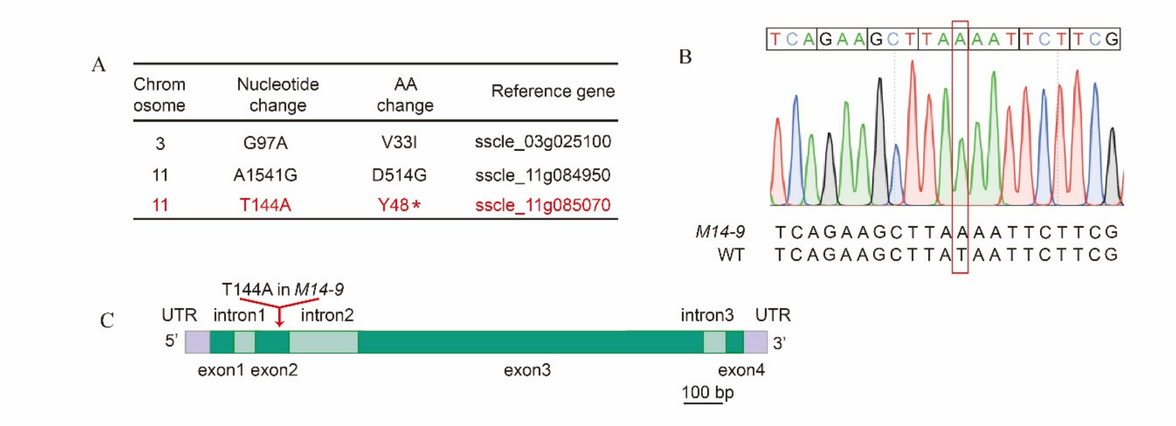

Figure S2: Identification of the molecular lesions of the M14-9 mutants.

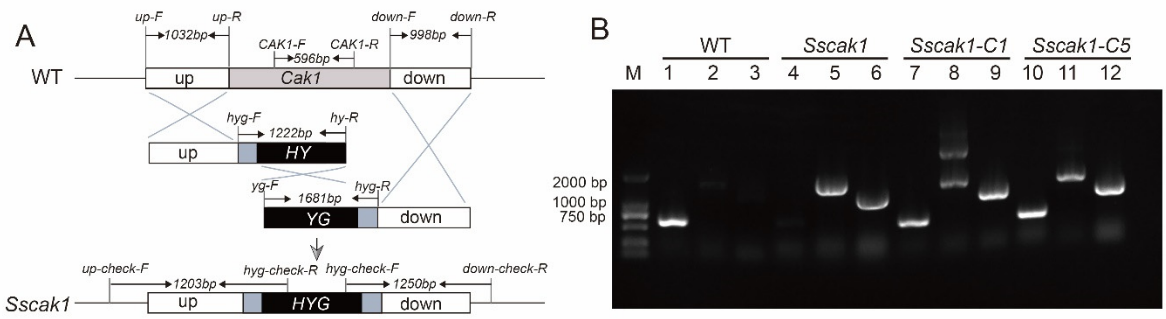

Figure S3: Identification of Sscak1 by PCR.

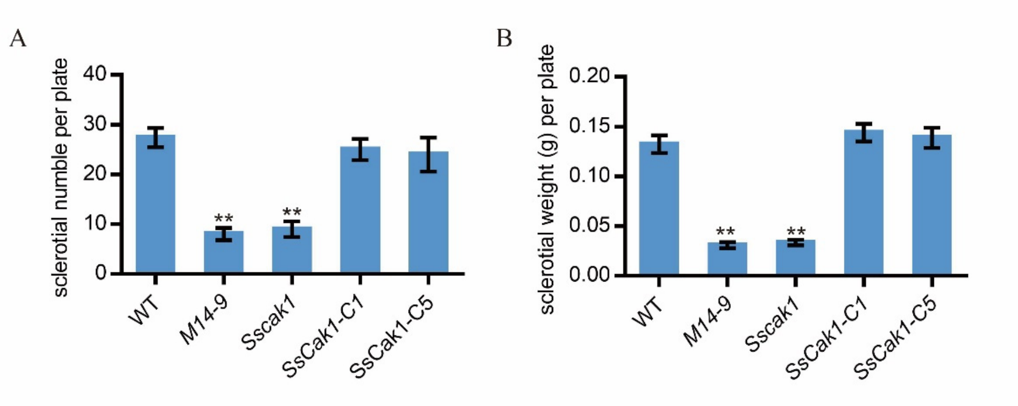

Figure S4: Sclerotia number and weight of SsCak1 knockout and complementation strains.

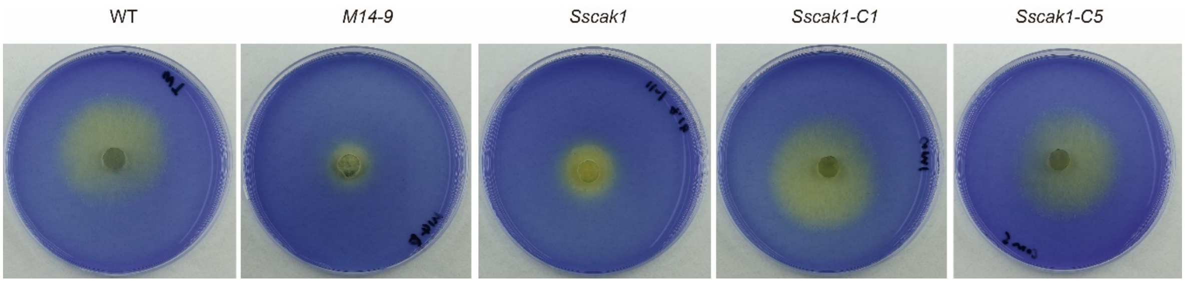

Figure S5: WT, M14-9, Sscak1, Sscak1-C1 and Sscak1-C5 grown on PDA medium containing bromophenol blue. Experiments were conducted three times with similar results.

Supplementary Table S1 Primer sequences used in this study.

up-F	tatgaccatgattacgaattc TCTGCGACCGAAGGATTACT
up-R	ccttcaatatcatcttct ATTGTCGAACCAAGAGGAT
down-F	tagaggtaatccttcttt CCAGGTCAAGTAATGTGTCT
down-R	tctagaggatccccgggtacc TTACGAATGTGTGGAGGTT
hyg-F	TGACTGGAGCGAGGCGATGT
hy-R	tctagaggatccccgggtaccGCATCATCGAAATTGCCGTCAACC
yg-F	tatgaccatgattacgaattcTCTCGGAGGGCGAAGAATCTCGTGC
hyg-R	AAAGAAGGATTACCTCTAAACAAGTGT
up-check-F	TTCATTGTTGACCTCCACTA
hyg-check-R	TACAGGACACACATTCATCG

hyg-check-F	TTATCCTCTTCACACGCTCG
down-check-R	TCTCCTTCTACATCCGCATC
CAK1-F	GAGATTGGTCTTGCTGTTGC
CAK1-R	CAGCATCTGGAAGTAACTCT
Cak1-RT-F	GCTACCTACGATGATTTATGC
Cak1-RT-R	ATTATGGAAGGATGCGAGAG
Sstub1-RT-F	GTGAGGCTGAGGGCTGTGTGA
Sstub1-RT-R	CCTTTGGCGATGGGACG
TRV-Cak1-F	gtgagtaaggttaccgaattcATGGACTCCACAACAGACTG
TRV-Cak1-R	gagacgcgtgagctcggtaccAACAGCGCCGCTAGAGATAT
TRV-PDS-F	gtgagtaaggttaccgaattcGAACATATTGAGTCAAAAGGTG
TRV-PDS-R	gagacgcgtgagctcggtaccGCTTCTGCTGAAGAGCAGAT
TRV-GFP-F	gtgagtaaggttaccgaattcTGGTGAGCAAGGGCGAGGAG
TRV-GFP-R	gagacgcgtgagctcggtaccTGGACGTAGCCTTCGGGCAT
